# Incidence and risk factors for modic changes in the lumbar spine: a systematic review and meta-analysis

**DOI:** 10.3389/fendo.2025.1585552

**Published:** 2025-07-30

**Authors:** Zhenyu Cao, Mingtao Zhang, Jingwen Jia, Guangzhi Zhang, Lei Li, Zhili Yang, Feng Zheng, Xuewen Kang

**Affiliations:** ^1^ Department of Orthopaedics, Lanzhou University Second Hospital, Lanzhou, Gansu, China; ^2^ Orthopaedics Key Laboratory of Gansu Province, Lanzhou, Gansu, China; ^3^ Department of Orthopaedics, Qinghai Provincial People’s Hospital, Xining, Qinghai, China

**Keywords:** modic change, lumbar spine, lower back pain, meta-analysis, incidence, risk factor

## Abstract

**Background:**

Modic changes refer to bone marrow alterations beneath vertebral endplates and are potentially linked to infection, trauma, disc degeneration, scoliosis, and other pathological conditions. Systematic evaluations of their incidence and associated risk factors in the lumbar spine are lacking. This study aimed to analyze and evaluate the incidence and risk factors of Modic changes in lumbar spine disorders.

**Methods:**

A comprehensive systematic review was conducted using Web of Science, PubMed, Cochrane Library, and Embase databases. Eligible studies reported the incidence and associated risk factors of Modic changes in the lumbar spine. Data were extracted and systematically analyzed from the selected studies, and meta-analyses were conducted employing random or fixed effects models.

**Results:**

Twenty-five studies were included in the meta-analysis. The overall incidence of Modic changes was 35%. Six risk factors were identified and quantitatively assessed. Strong evidence supported the association of endplate changes (OR=3.56; 95% CI=2.00 to 6.32; p<0.0001); moderate evidence supported the association of age (OR=4.01; 95% CI=1.37 to 6.65; p=0.003), disc degeneration (OR=8.54; 95% CI=1.98 to 36.73; p=0.004), and lumbar lordosis angle (OR=-4.14; 95% CI=-6.79 to -1.49; p=0.002); minor evidence supported the association of spondylolisthesis (OR=2.00; 95% CI=1.12 to 3.58; p=0.02) and physical labor (OR=1.81; 95% CI=1.08 to 3.04; p=0.03) with the occurrence of Modic changes in the lumbar spine. No significant associations were found to support body mass index, sex, disc herniation, smoking, distributional segmentation, or sacral slope angle as risk factors for Modic changes in the lumbar spine.

**Conclusion:**

Modic changes occur in 35% of lumbar spine cases, with advanced age, disc degeneration, endplate changes, spondylolisthesis, reduced anterior lumbar lordosis angles, and participation in physical labor identified as associated risk factors.

## 1 Introduction

Lower back pain is a prevalent symptom of spinal disorders, often caused by various underlying conditions and associated with a certain disability rate. Severe disabling pain significantly impairs the long-term quality of life for patients and imposes a substantial burden on global economic development (1–4). The etiology of lower back pain is highly complex, involving factors such as lumbar disc degeneration, lumbar disc herniation, lumbar muscle strain, and lumbar spondylolisthesis (2, 5–8). Furthermore, Modic changes are thought to be strongly associated with lower back pain (9–11).

In 1987, De Roos reported Modic-like changes using nuclear magnetic resonance (NMR) imaging of the lumbar spine (12). Modic changes refer to alterations in the bony tissue beneath the vertebral endplates, which can be differentiated and confirmed via magnetic resonance imaging (MRI) (13). Modic changes are classified into three distinct types based on NMR manifestations: Type I is characterized by edema and inflammatory changes in the bone beneath the endplate; Type II is marked by fatty infiltration and degenerative changes; Type III is distinguished by sclerotic alterations in the local bone beneath the endplate (14–17). Modic changes are significantly associated with severe disc herniation, disc degeneration, and chronic lower back pain (12, 17–19). Nevertheless, their pathogenesis remains complex, and their etiology is yet to be fully elucidated. This may be related to compromised defensive properties, such as the structural integrity of the vertebral endplates, along with other contributing factors (20).

Numerous studies have investigated risk factors associated with Modic changes. Li et al. (21) demonstrated that factors such as disc degeneration, spondylolisthesis, and disc height may be linked to Modic changes. Some studies have suggested that obesity and elevated body mass index (BMI) may be associated with Modic changes (22), while others have found no such relationship (23). Furthermore, whether smoking, lumbar disc herniation, and lumbar segmental distribution are associated with the occurrence and progression of Modic remains controversial. To date, no meta-analysis has been conducted on the incidence and associated risk factors of Modic changes in the lumbar spine. Comprehensive analyses are crucial for confirming these factors, as they are essential for the early diagnosis and management of degenerative spinal conditions. Therefore, this systematic review and meta-analysis aimed to determine the incidence and risk factors of Modic changes in patients with lumbar spinal disorders.

## 2 Methods

This systematic review and meta-analysis adhered to the guidelines outlined in the Preferred Reporting Items for Systematic Reviews and Meta-Analyses (PRISMA) statement (24) and AMSTAR (Assessing the Methodological Quality of Systematic Reviews) Guidelines (25).

### 2.1 Literature search strategies

A comprehensive search of all relevant English-language publications available as of May 10, 2024, was conducted across the PubMed, Embase, Web of Science, and Cochrane Library databases, in consultation with independent information technology experts. The search strategy (**Table 1**) utilized the following search terms: disc degeneration, lumbar spine, lower back pain, Modic changes, incidence, and risk factors. Titles and abstracts were screened to identify potentially relevant articles, and full-text publications and reference lists were obtained for further in-depth analysis based on the inclusion criteria.

**Table 1:** Search strategy keywords.

Concept	Keywords used in the strategy
Intervertebral disc degeneration	(“lumbar spine*”) OR (“lumbar vertebra*”) OR (“Intervertebral Disk Degeneration*”) OR (“Intervertebral Disc Degeneration*”) OR (“intervertebral disk*”) OR (“intervertebral disc*”) OR (“disc herniat*”) OR (“disk herniat*”) OR (“disc disease*”) OR (“disk disease*”) OR (“low back pain”)OR (“Lumbago”)OR (“Low Back Ache”)OR (“Low Backache”)OR (“Postural Low Back Pain”)OR (“Recurrent Low Back Pain”)OR (“Mechanical Low Back Pain”)
Modic change	"Modic change" OR "Modic changes"
Risk	Risk* OR factor* OR prevalen* OR predict*OR Social Risk Factor* OR Health Correlates* OR incidence* OR “odds ratio”* OR “Relative odds”* OR “frequency”OR “morbidity”OR “epidemiology”OR “Social Epidemiology”OR “Cross-Product Ratios”OR “surveillance”OR “occurrence”OR “outbreaks”OR “endemics”

*Indicates truncation of search term.

### 2.2 Inclusion and exclusion criteria

Studies that met the following inclusion criteria were included for review: (1) Patients of all ages suffering from low back pain due to degenerative lumbar spine conditions (including lumbar disc herniation, lumbar spondylolisthesis, and degenerative lumbar lateral herniation, among other degenerative lumbar spine conditions), treated surgically or non-surgically; (2) Healthy participants; (3) Modic changes beneath the vertebral endplates confirmed by MRI; and (4) Publications in English language. Studies were excluded based on the following: (1) Pregnancy, traumatic fracture, spinal infection or tuberculosis, spinal deformity, malignancy, radiotherapy, recent use of antibiotics or corticosteroids, and psychological or psychiatric disorders; (2) Meta-analyses, case reports, reviews, technical notes, or abstracts only; and (3) Missing clinical data.

### 2.3 Quality assessment

Two independent reviewers used the Newcastle-Ottawa Scale (NOS) as a tool to assess the quality of the included studies, which were evaluated based on patient selection, group comparability, and the identification of outcomes of interest. Study quality was rated on a scale of 0 to 9, with low (1–3), medium (4–6), and high (7–9) ratings. Any discrepancy between the reviewers was resolved through consensus following discussion.

### 2.4 Data extraction

Two independent reviewers screened all titles and abstracts, selecting relevant articles for full-text review. Data were extracted from all eligible studies that met the inclusion criteria, including information on authorship, publication date, country of origin, patient type, sample size per factor, incidence of Modic changes, study design, and level of evidence. In cases where data were missing or unavailable, the corresponding authors were contacted. If the authors were unable to provide the necessary data, the study was excluded from the analysis.

### 2.5 Statistical analysis

Meta-analysis was performed on studies investigating the incidence of Modic changes in the lumbar spine and the associated risk factors. Statistical analyses and graphical representations were generated using Review Manager software (RevMan) version 5.4. Studies reporting odds ratios (ORs) were included in the meta-analysis. Heterogeneity was assessed using the I² statistic, with slight statistical heterogeneity defined as I² < 50%. Data were analyzed using a fixed-effects model, or a random-effects model if I² ≥ 50%. The estimated incidence was calculated as a combined proportion with a 95% confidence interval (CI).

## 3 Results

### 3.1 Search results

**Figure 1:**
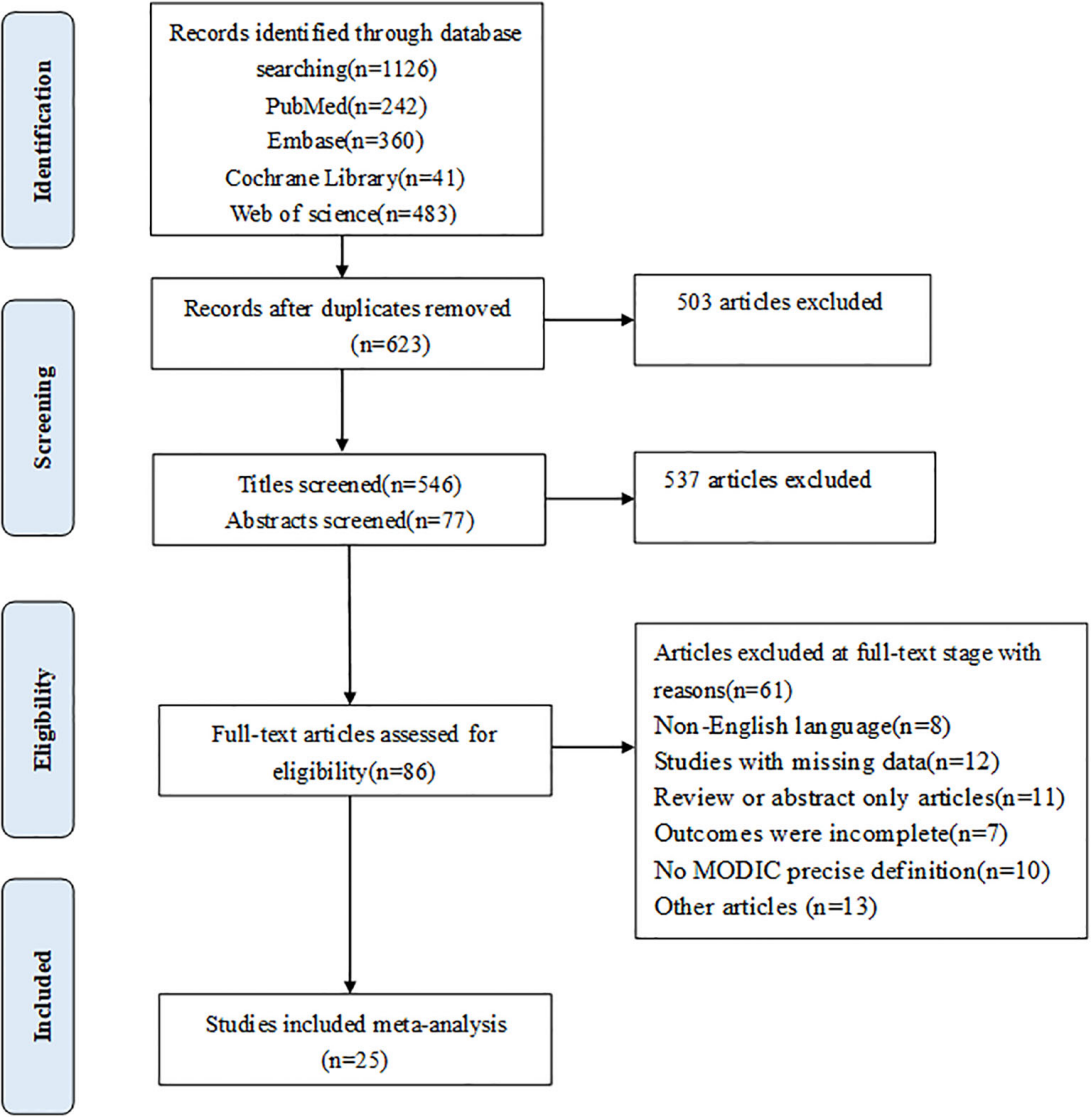
Search result.

The study initially retrieved a total of 1,126 articles for review. Most of the articles were excluded based on the predefined inclusion criteria. Titles and abstracts were screened, and 86 potentially relevant papers were selected for further assessment (**Figure 1**). Ultimately, 25 studies met the inclusion criteria.

### 3.2 Characteristics of included studies

The 25 studies that met the inclusion criteria encompassed 10,440 individuals, including patients with low back pain and healthy participants aged 10–89 years. Of these studies, 4 were prospective, 16 were retrospective, and 8 were cross-sectional in design. Nineteen studies were conducted in Asia (twelve in China (9, 20, 21, 23, 26–33), five in Turkey (22, 34–37), one in India (38), one in Japan (39)), four in Europe (two in Denmark (40, 41), one in Switzerland (42), one in the Netherlands (43)), and two in the Americas (all in the U.S.A (44, 45).). The mean quality score obtained from the included studies based on the NOS criteria was 6.84 ± 0.80. According to the level of evidence ratings, seven studies were classified as level II, nine as level III, and nine as level IV (**Table 2**). According to the GRADE criteria, seven risk factors received high-level recommendations, whereas five were classified as moderate. The overall incidence of Modic changes in the lumbar spine across the studies was 35%, with 12 identified and quantified risk factors. The characteristics of the studies and patient demographics are presented in **Table 2**.

**Table 2:** The characteristics of the studies and patient demographics.

Lead author (Year)	Location	Age (mean±sd)	Gender (male%)	No. of total patient	No. of Modic change	Type of patient	Method	Study design	Level of evidence	NOS
Khaled Aboushaala (2024)	USA	56.9±15.7	58.1%	50	13	Symptomatic intervertebral disc degeneration or lumbar disc herniation	Modic and no modic analyse	Prospective Cross-sectional	IV	7
Guang-qing Li (2024)	China	NR	33.7%	719	150	Spondylolisthesis	Modic and no modic analyse	Retrospective	IV	6
İlter Demirhan (2023)	Turkey	NR	NR	90	45	Low back pain	Modic and no modic analyse	Prospective	III	8
Beixing Wei (2023)	China	NR	44.0%	500	126	Low back pain	Modic and no modic analyse	Retrospective	III	6
Yang Xiao (2023)	China	NR	39.0%	954	66	Lumbar degenerative diseases	Modic and no modic analyse	Retrospective	IV	6
Peter M. Udby (2024)	Denmark	NR	50.2%	620	290	Disc herniation	Modic and no modic analyse	Retrospective Cross-sectional	II	8
G. Michael Mallow (2022)	USA	16.5±2.4	46.9%	207	29	Low back pain	Modic and no modic analyse	Retrospective	IV	6
Emel Ece Özcan-Eksi (2021)	Turkey	NR	33.8%	151	56	Low back pain	Modic and no modic analyse	Retrospective	III	7
Emel Ece Ozcan-Eksi (2021)	Turkey	36.33±6.9	45.9%	527	132	Low back pain	Modic and no modic analyse	Retrospective Cross-sectional	III	6
Xiaoping Mu (2020)	China	NR	52.0%	1019	138	Low back pain	Modic and no modic analyse	Retrospective	III	6
Murat S.akir Eksi (2019)	Turkey	61.27±13.74	50.0%	3999	33	Low back pain	Modic and no modic analyse	Cross-sectional	IV	8
Ibrahim Burak Atci (2020)	Turkey	NR	60.9%	141	61	Low back pain	Modic and no modic analyse	Retrospective	IV	8
Stefan Dudli (2020)	Switzerland	NR	51.9%	54	21	Low back pain	Modic and no modic analyse	Cross-sectional	II	7
Peter van der Wurff (2019)	Netherlands	NR	90.2%	286	60	Low back pain	Modic and no modic analyse	Retrospective	II	7
Mindong Lan (2019)	China	NR	NR	277	62	Low back pain	Modic and no modic analyse	Retrospective	IV	7
Lunhao Chen (2018)	China	53.6±14.9	41.6%	442	209	Participant	Modic and no modic analyse	Cross-sectional	II	6
Runsheng Guo (2018)	China	54.31±13.9	52.6%	293	NA	Hospitalised patients	Modic and no modic analyse	Retrospective	III	8
Long Xiao (2017)	China	NR	58.3%	345	120	Low back pain	Modic and no modic analyse	Retrospective	II	6
Chao Han (2017)	China	NR	47.1%	230	47	Low back pain	Modic and no modic analyse	Retrospective	IV	7
Rishi Mugesh Kanna (2017)	India	36.7±10.8	56.2%	809	107	Spine patients	Modic and no modic analyse	Prospective	III	8
Florence P.S. Mok (2016)	China	40.4±10.9	NR	2449	141	Participant	Modic and no modic analyse	Cross-sectional	III	6
Juhani H. Ma'aitta (2016)	China	52.9±6.5	37.2%	1546	282	Participant	Modic and no modic analyse	Cross-sectional	IV	6
Tetsuo Hayashi (2015)	Japan	44.6±12.2	59.3%	450	129	Low back pain	Modic and no modic analyse	Retrospective	II	7
Hai-Long Wu (2012)	China	NR	39.2%	209	92	DLS patients	Modic and no modic analyse	Retrospective	II	7
Tue Secher Jensen (2010)	Denmark	NR	47%	412	200	Low back pain	Modic and no modic analyse	Prospective	III	7

### 3.3 Incidence of modic changes

**Figure 2:**
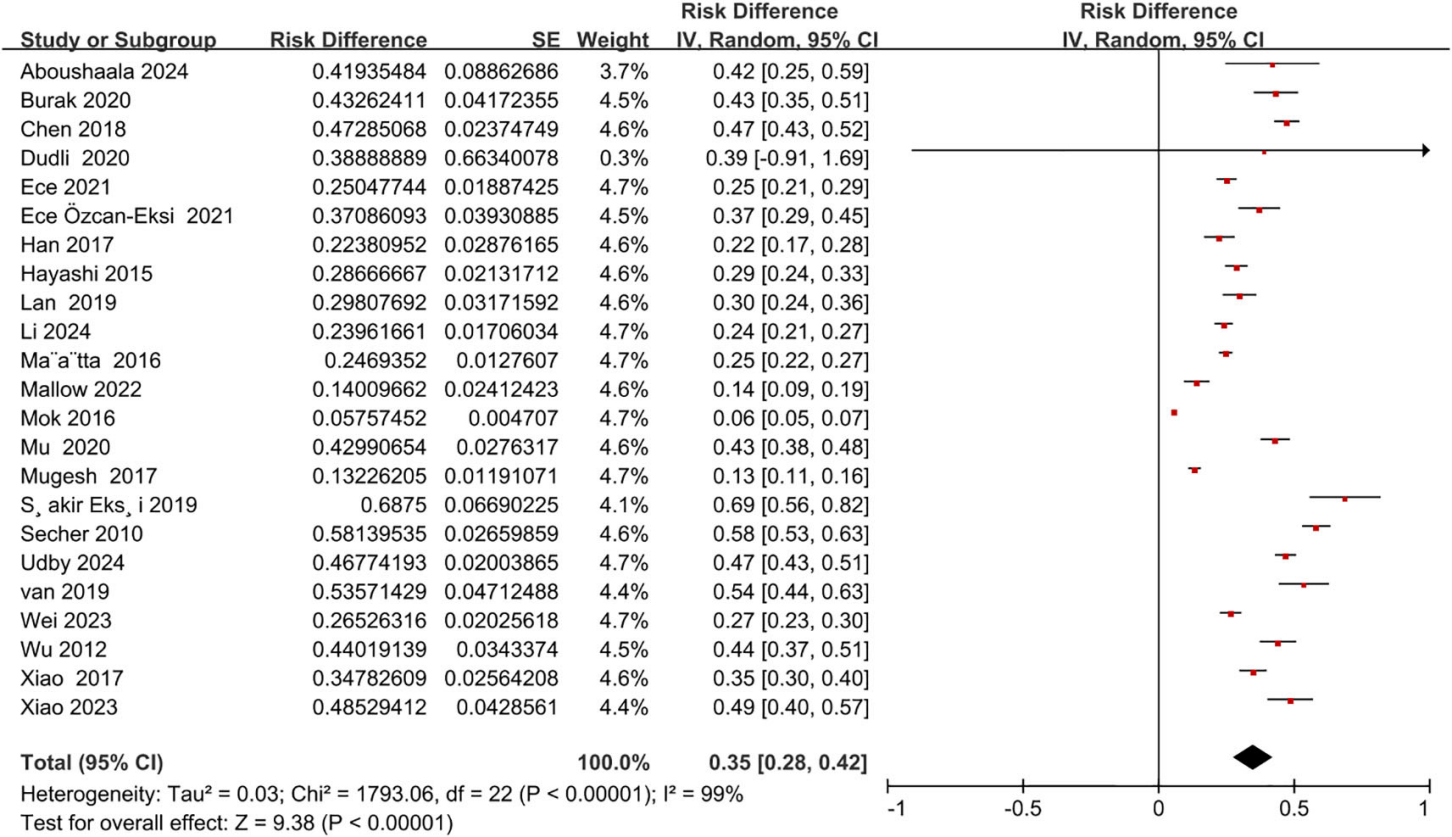
The overall incidence of Modic changes.

This meta-analysis pooled data from 25 studies, encompassing a total of 10,440 patients and healthy participants. The overall incidence of Modic changes in the lumbar spine was 35% (95% CI = 0.28 to 0.42; Z = 9.38; p < 0.00001; I² = 99%) (**Figure 2**).

### 3.4 Risk factors

Seventeen risk factors were identified, of which 12 were quantitatively analyzed based on the 25 studies included in the review. The risk factors identified in more than three studies included sex, age, smoking, BMI, disc degeneration, lumbar disc herniation, endplate changes, anterior lumbar lordosis angle, sacral angle, spondylolisthesis, segmental distribution, and physical labor.

### 3.5 Patient-related factors

#### 3.5.1 Age

**Figure 3:**
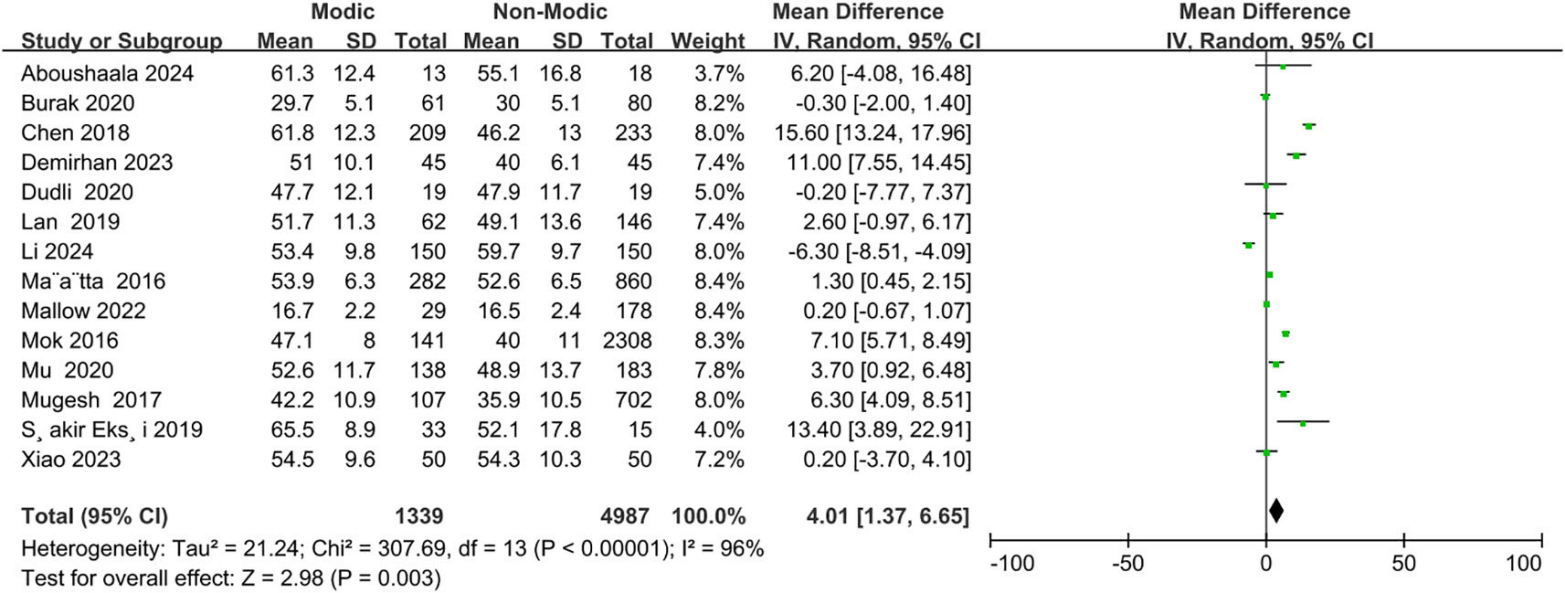
Age as a risk factor.

A total of 14 studies (9, 21, 23, 26, 29, 30, 33, 34, 36–38, 42, 44, 45) reported an association between age and the occurrence of Modic changes (**Figure 3**). The meta-analysis demonstrated that older patients were more likely to develop Modic changes than younger patients (OR = 4.01; 95% CI: 1.37 to 6.65; Z = 2.98; p = 0.003; I² = 96%; **Figure 3**). Based on the reviewed studies, we found moderate evidence suggesting older age as an important risk factor for Modic changes in the lumbar spine.

#### 3.5.2 Physical labor

**Figure 4:**
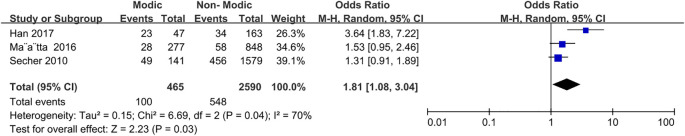
Physical labor as a risk factor.

Three studies (32, 33, 41) reported correlations between physical labor and the occurrence of Modic changes (**Figure 4**). Analysis of the pooled data indicated that patients who engaged in physical labor were more likely to develop Modic changes. The meta-analysis revealed that Modic changes occurred in 15.43% of patients who engaged in physical labor and in 15.16% of patients who did not (OR = 1.81; 95% CI: 1.08 to 3.04; Z = 2.23; p = 0.03; I² = 70%; **Figure 4**). Therefore, we found minor evidence suggesting that engagement in physical labor is a risk factor for the development of Modic changes.

#### 3.5.3 Sex, BMI, and smoking

**Figure 5:**
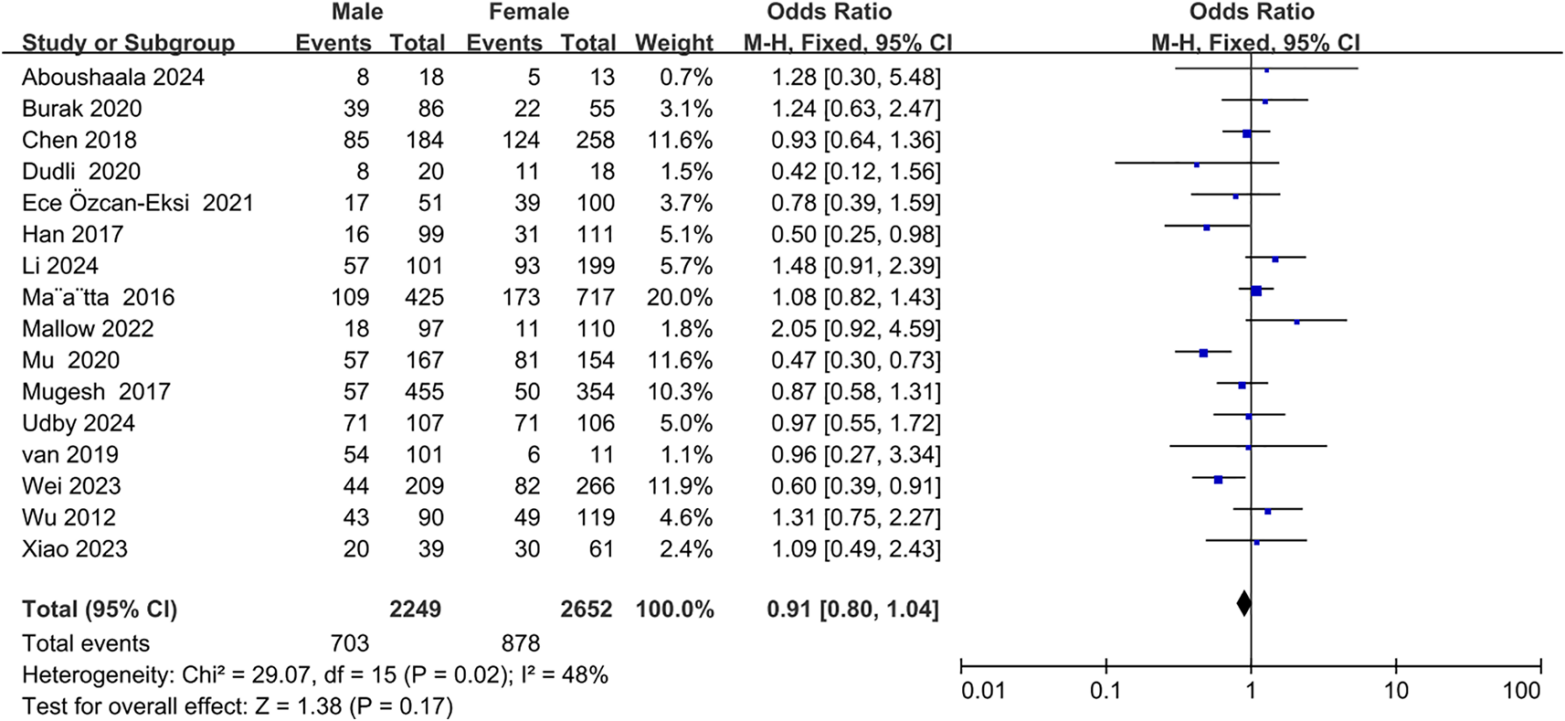
Gender as a risk factor.

**Figure 6:**
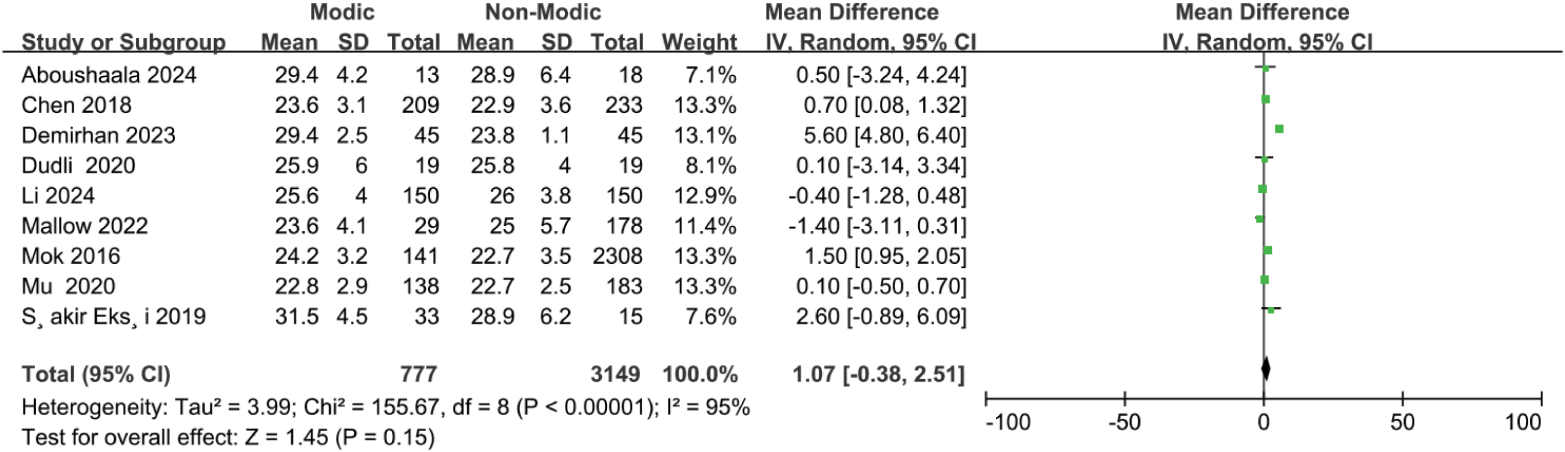
Body mass index (BMI) as a risk factor.

**Figure 7:**
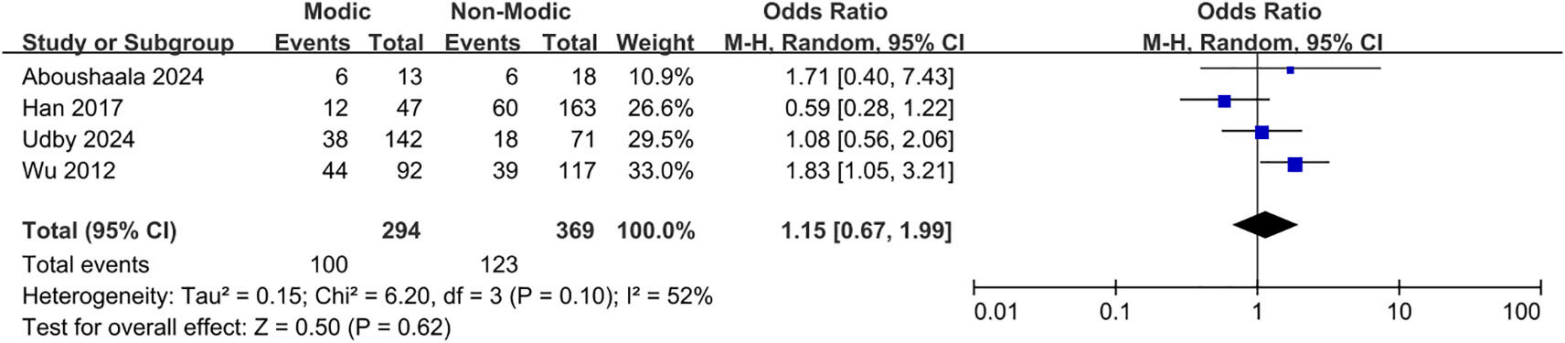
Smoking as a risk factor.

Sixteen studies (20–23, 26, 27, 30, 32, 33, 37, 38, 40, 42–45) demonstrated a correlation between sex and Modic changes, indicating that sex was not significantly associated with the occurrence of Modic changes (OR = 0.91; 95% CI = 0.80 to 1.04; Z = 1.38; p = 0.17; I² = 48%; **Figure 5**). Additionally, nine studies (9, 21, 23, 30, 34, 36, 42, 44, 45) investigated the relationship between BMI and Modic changes, and no significant correlation was found (OR = 1.07; 95% CI = -0.38 to 2.51; Z = 1.45; p = 0.15; I² = 95%; **Figure 6**). Four studies (27, 32, 40, 44) confirmed no significant correlation between smoking and the presence of Modic changes (OR = 1.15; 95% CI = 0.67 to 1.99; Z = 0.50; p = 0.62; I² = 52%; **Figure 7**). Consequently, this meta-analysis confirmed that sex, body mass index, and smoking were not significant risk factors for Modic changes in the lumbar spine.

### 3.6 Imaging-related factors

#### 3.6.1 Degenerative discs

**Figure 8:**
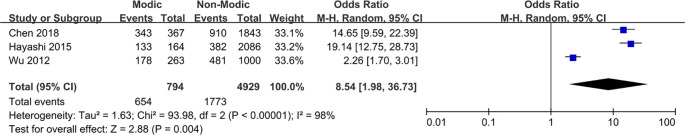
Disc degeneration as a risk factor.

Three studies (27, 30, 39) reported an association between disc degeneration and Modic changes (**Figure 8**). The pooled data analysis indicated a correlation between the presence of disc degeneration and the occurrence of Modic changes in patients. The meta-analysis revealed that Modic changes occurred in 26.95% of patients with disc degeneration and 4.25% of patients without disc degeneration (OR = 8.54; 95% CI: 1.98 to 36.73; Z = 2.88; p = 0.004; I² = 98%; **Figure 8**). Consequently, this analysis provides moderate evidence that disc degeneration is an important risk factor for Modic changes in the lumbar spine.

#### 3.6.2 Endplate changes

**Figure 9:**
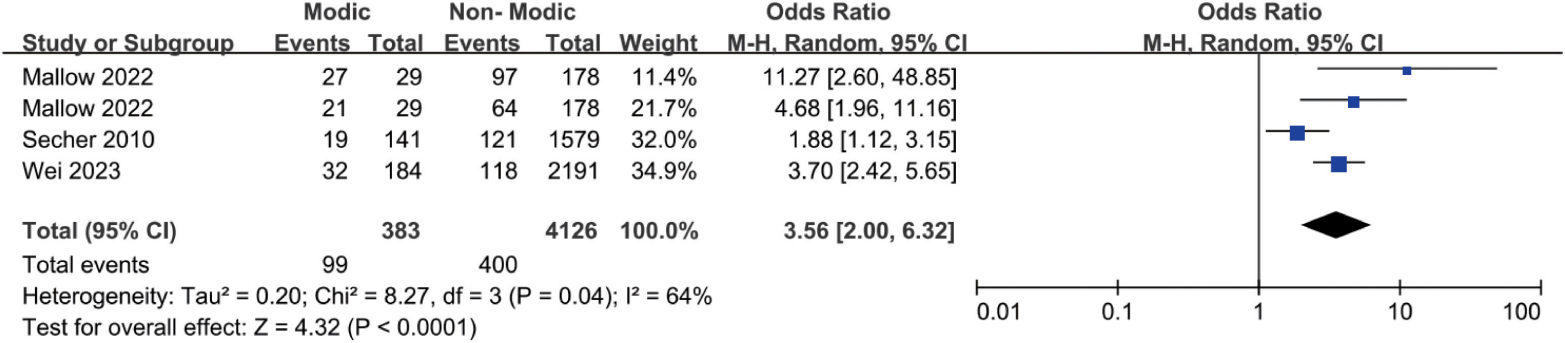
Endplate changes as a risk factor.

Three studies (20, 41, 45) reported an association between endplate changes and Modic changes (**Figure 9**). The pooled data analysis indicated a correlation between endplate changes and the occurrence of Modic changes in patients. The meta-analysis revealed that 19.84% of patients with endplate alterations developed Modic changes, compared to 7.08% of those without endplate alterations (OR = 3.56; 95% CI = 2.00 to 6.32; Z = 4.32; p < 0.0001; I² = 64%; **Figure 9**). Consequently, this analysis provides compelling evidence that endplate changes represent a key risk factor for Modic changes in the lumbar spine.

#### 3.6.3 Lumbar lordosis angle

**Figure 10:**
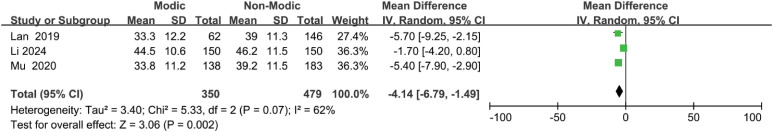
Lumbar lordosis angle as a risk factor.

Three studies (21, 23, 29) reported an association between lumbar lordosis and Modic changes (**Figure 10**). The pooled data analysis indicated that patients with smaller anterior lumbar lordosis angles were more likely to exhibit Modic changes. The meta-analysis revealed that patients with smaller anterior lumbar lordosis angles were more likely to develop Modic changes, while those with larger anterior lumbar lordosis angles were less likely to do so (OR = -4.14; 95% CI = -6.79 to -1.49; Z = 3.06; p = 0.002; I² = 62%; **Figure 10**). Based on these results, we found moderate evidence suggesting that a reduced anterior lumbar lordosis angle appears to be an important risk factor for Modic changes in the lumbar spine.

#### 3.6.4 Spondylolisthesis

**Figure 11:**
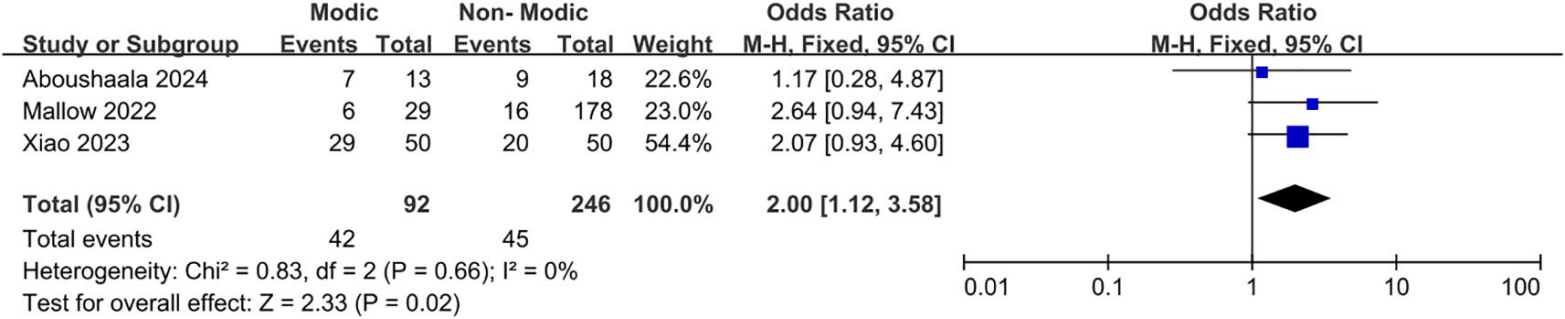
Spondylolisthesis as a risk factor.

Three studies (26, 44, 45) reported an association between spondylolisthesis and Modic changes (**Figure 11**). Pooled data analysis indicated that patients with spondylolisthesis were more likely to develop Modic changes, while the meta-analysis revealed that Modic changes occurred in 48.28% of patients with spondylolisthesis and 19.92% of those without spondylolisthesis (OR = 2.00; 95% CI = 1.12 to 3.58; Z = 2.33; p = 0.02; I² = 0%; **Figure 11**). Therefore, we found minor evidence suggesting that spondylolisthesis is a risk factor for Modic changes in the lumbar spine.

#### 3.6.5 Lumbar disc herniation, segmental distribution, and sacral slope angle

**Figure 12:**
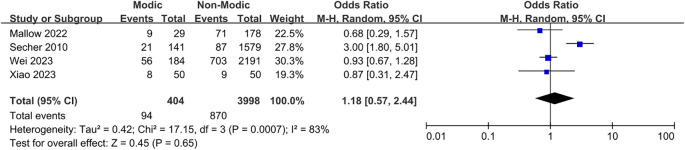
Lumbar disc herniation as a risk factor.

**Figure 13:**
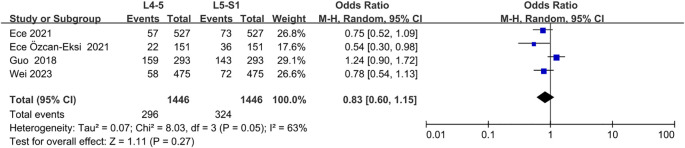
Segmental distribution as a risk factor.

**Figure 14:**
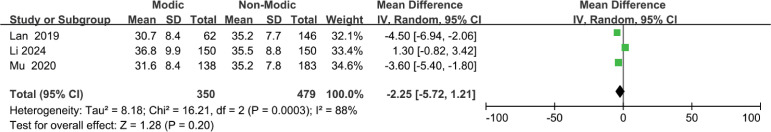
Sacral slope angle as a risk factor.

Four studies (20, 26, 41, 45) investigated the correlation between lumbar disc herniation and Modic changes, indicating that lumbar disc herniation was not significantly associated with the presence of Modic changes (OR = 1.18; 95% CI = 0.57 to 2.44; Z = 0.45; p = 0.65; I² = 83%; **Figure 12**). Additionally, four studies (20, 22, 31, 35) reported no significant associations between segmental distribution and Modic alterations (OR = 0.83; 95% CI = 0.60 to 1.15; Z = 1.11; p = 0.27; I² = 63%; **Figure 13**). Furthermore, three studies (21, 23, 29) confirmed that the size of the sacral slope angle was not significantly associated with the presence of Modic alterations (OR = -2.25; 95% CI = -5.72 to 1.21; Z = 1.28; p = 0.20; I² = 88%; **Figure 14**). Therefore, this meta-analysis found no substantial evidence to confirm that lumbar disc herniation, segmental distribution, or sacral slope angle are significant risk factors for the development of Modic changes in the lumbar spine.

## 4 Discussion

This systematic review and meta-analysis yielded three key findings. First, the analysis determined a 35% incidence of Modic changes in the lumbar spine based on data from X studies. Second, strong evidence suggests that the risk factors associated with the development of Modic changes in the lumbar spine include advanced age, lumbar disc degeneration, endplate changes, spondylolisthesis, reduced anterior lumbar lordosis angles, and participation in physical labor. Third, no evidence was found linking sex, BMI, smoking, lumbar disc herniation, segmental distribution, or sacral angle to Modic changes in the lumbar spine.

Lower back pain associated with Modic changes, characterized by abnormal alterations in the vertebral endplates and sub-endplates, has garnered significant research attention. These changes appear as abnormal signals in the endplates on MRI. Modic changes can induce disabling lower back pain (46, 47). The present study demonstrated an incidence rate of 35% for Modic changes, higher than that in the general population, possibly due to the broader population range included in this study. However, since De Roos et al.’s initial study (12), research on its pathogenesis and risk factors has remained controversial. This meta-analysis provides robust evidence demonstrating an association between Modic changes and various factors, including age, disc degeneration, endplate changes, spondylolisthesis, the anterior lumbar lordosis angle, and physical labor.

The 14 studies included in this meta-analysis indicated that patients with Modic changes were generally older, consistent with most previous findings (9, 23, 30, 33, 36). However, Li et al. (21) reported that in patients with lumbar spondylolisthesis, Modic changes were more prevalent in younger individuals (mean age, 53.44 ± 9.80 years; n = 150) than in older individuals (mean age, 59.68 ± 9.66 years; n = 150). Mallow et al. (45) reported that Modic changes were present in a subset of adolescents (Modic group: 16.7 ± 2.2 years, n = 29; non-Modic group: 16.5 ± 2.4 years, n = 178). A focus on Modic changes in younger patients may hold significant potential for advancing our understanding of its pathogenesis and providing treatment strategies. Additionally, some researchers have reported no significant relationship between age and the development of Modic changes (26, 29, 42, 44). Definitive evidence from this meta-analysis suggests that older patients are more prone to Modic changes, which may be linked to the development of disc degeneration, spondylolisthesis, reduced vertebral stability, spinal stenosis, and osteoporosis; however, the specific causes and mechanisms underlying these associations remain unclear.

Studies consistently report that disc degeneration has the potential to trigger Modic changes. Eksi et al. (35) investigated the relationship between severe disc degeneration and Modic changes, concluding that this association is particularly strong in the lower lumbar region, with Modic changes detected in 88–90% of cases involving disc degeneration in this area. Wu et al. (27) identified disc degeneration as a risk factor for Modic changes, suggesting that patients with disc degeneration (27.0% [178/659]) are more likely to develop Modic changes than those without disc degeneration (14.1% [85/604]). Jensen et al. (41) found a strong correlation between disc degeneration and new-onset Modic changes, suggesting that disc degeneration may be a potential predictor for Modic alterations. The current meta-analysis of three studies found strong evidence of a significant correlation between disc degeneration and Modic changes, with a higher incidence of Modic changes in patients with disc degeneration (26.95% [654/2427]) than in those without (4.25% [140/3296]). This may be related to changes in angular motion, translational motion, and disc height in degenerated discs. However, the exact mechanism remains unclear, warranting further research to confirm this association.

Endplate changes included Schmorl’s nodes and endplate damage. The current meta-analysis, comprising three studies, demonstrated a higher incidence of Modic changes in patients with endplate changes (19.84% [99/499]) than in those without (7.08% [284/4010]). Jensen et al. (41) concluded that endplate changes were strongly associated with Modic changes, as indicated by a higher incidence of Modic changes in patients with endplate changes (13.57% [19/140]) than in those without (7.72% [122/1580]), suggesting that endplate changes could serve as a potential predictor. Similar findings were reported by Wei et al. (20), in which the incidence of Modic changes was higher in patients with endplate changes (21.33% [32/150]) than in those without endplate changes (6.83% [152/2225]). Mallow et al. (45) found a strong correlation between endplate changes and the presence of Modic changes, with the incidence of Modic changes being higher in patients with endplate changes (endplate damage, 21.8% [27/124]; Schmorl’s nodes, 24.71% [21/85]) than in those without endplate changes (endplate damage, 2.4% [2/83]; Schmorl’s nodes, 6.56% [8/122]). These studies suggest that the association between endplate changes and Modic changes is becoming increasingly evident. This association may be due to endplate changes that cause microstructural alterations in the disc and microfractures in the endplate, potentially leading to bacterial invasion. However, the specific mechanisms through which endplate changes influence Modic changes remain unclear and require further investigation.

Spondylolisthesis, physical labor, and anterior lumbar lordosis angles warrant attention. Wei et al. (20) found a strong correlation between lumbar spondylolisthesis and the occurrence of Modic changes, with a higher incidence in patients with spondylolisthesis (32.7% [32/98]) than in those without (6.7% [152/2277]); most Modic changes were characterized as Modic type II. Hayashi et al. (39) demonstrated that the incidence of Modic changes in patients with a spondylolisthesis degree greater than 0.8 mm (10.9% [119/1082]) was higher than in those with a spondylolisthesis degree less than or equal to 0.8 mm (3.85% [45/1168]), providing strong evidence of the correlation between spondylolisthesis severity and Modic changes. In study, Modic type II was identified as the most prevalent form of Modic change, and Modic change was more common in patients with spondylolisthesis (45). This is hypothesized to be associated with bone marrow changes and the disease’s developmental stage (48). Han et al. (32) concluded that the incidence of Modic changes was higher in patients who engaged in heavier physical labor (40.4% [23/57]) than in those who engaged in lighter physical labor (15.7% [24/153]). Additionally, Modic type III changes were more prevalent in patients who performed more rigorous physical labor, suggesting that physical labor can be considered a risk factor (32). This may be related to microinjuries of the vertebral endplates and fatigue degeneration caused by heavy physical labor, consistent with the findings of previous studies (49, 50). The anterior lumbar lordosis angle is a distinctive angle that has evolved in humans to maintain an upright posture (51). Xia et al. (52) concluded that the frequency of Modic changes was negatively correlated with the anterior lumbar lordosis angle, potentially owing to axial decompression of the vertebral body and alterations in endplate shear. However, Li et al. (21) found no significant correlation between Modic changes and the anterior lumbar lordosis angle. This meta-analysis demonstrated that spondylolisthesis, physical labor, and the anterior lumbar lordosis angle are strongly associated with Modic changes. Patients with spondylolisthesis exhibited a higher incidence of Modic changes (48.28% [42/87]) than those without spondylolisthesis (19.92% [50/251]). Similarly, individuals who engaged in physical labor had a higher incidence of Modic alterations (15.43% [100/648]) than those who did not engage in physical labor (15.16% [365/2407]). Additionally, patients with smaller anterior lumbar lordosis angles were more likely to exhibit Modic changes. These factors can be considered risk factors for Modic changes.

This review and meta-analysis had some limitations. Although 25 studies were included, their overall methodological quality was moderate, and the inclusion of lower-quality studies may have influenced the results. Subtype-specific analyses of Modic changes (Types I–III) were not conducted due to the insufficient data available from the included studies. Furthermore, significant heterogeneity was observed, indicating considerable inter-study variability. Future research should prioritize high-quality studies and employ more advanced methodologies.

## 5 Conclusion

The objective of this systematic review and meta-analysis was to determine the incidence and associated risk factors of Modic changes in the lumbar spine. The findings suggest an approximate incidence of 35%, with risk factors including advanced age, disc degeneration, endplate changes, spondylolisthesis, reduced anterior lumbar lordosis angles, and participation in physical labor.

## Data Availability

The raw data supporting the conclusions of this article will be made available by the authors, without undue reservation.
